# DRPPM-EASY: A Web-Based Framework for Integrative Analysis of Multi-Omics Cancer Datasets

**DOI:** 10.3390/biology11020260

**Published:** 2022-02-08

**Authors:** Alyssa Obermayer, Li Dong, Qianqian Hu, Michael Golden, Jerald D. Noble, Paulo Rodriguez, Timothy J. Robinson, Mingxiang Teng, Aik-Choon Tan, Timothy I. Shaw

**Affiliations:** 1 Department of Biostatistics and Bioinformatics, H. Lee Moffitt Cancer Center, Tampa, FL 33612, USA; alyssa.obermayer@moffitt.org (A.O.); Mingxiang.Teng@moffitt.org (M.T.); AikChoon.Tan@moffitt.org (A.-C.T.); 2 Computational Biology Department, St Jude Children’s Research Hospital, Memphis, TN 38105, USA; li.dong@stjude.org; 3 Department of Drug Discovery, Moffitt Cancer Center, Tampa, FL 33612, USA; Qianqian.Hu@moffitt.org; 4 University of Central Florida, Orlando, FL 32816, USA; michaelgolden00true@gmail.com; 5 Department of Radiation Oncology, Moffitt Cancer Center, Tampa, FL 33612, USA; Jerald.Noble@moffitt.org (J.D.N.); Timothy.Robinson@moffitt.org (T.J.R.); 6 Department of Immunology, Moffitt Cancer Center, Tampa, FL 33612, USA; Paulo.Rodriguez@moffitt.org

**Keywords:** R Shiny application, RNA-seq, proteomics, multi-omics analysis, T-cell acute lymphoblastic leukemia, CCLE

## Abstract

**Simple Summary:**

With the influx of multi-omics profiling, effective integration of these data remains the bottleneck for omics-driven discovery. Thus, we developed DRPPM-EASY, an R Shiny framework for integrative multi-omics analysis of cancer datasets. Our tool enables the exploration of multi-omics data by providing a simple user interface that minimizes the need for computational experience. Furthermore, the interface can be deployed locally or on a webserver to facilitate scientific collaboration and discovery.

**Abstract:**

High-throughput transcriptomic and proteomic analyses are now routinely applied to study cancer biology. However, complex omics integration remains challenging and often time-consuming. Here, we developed DRPPM-EASY, an R Shiny framework for integrative multi-omics analysis. We applied our application to analyze RNA-seq data generated from a USP7 knockdown in T-cell acute lymphoblastic leukemia (T-ALL) cell line, which identified upregulated expression of a TAL1-associated proliferative signature in T-cell acute lymphoblastic leukemia cell lines. Next, we performed proteomic profiling of the USP7 knockdown samples. Through DRPPM-EASY-Integration, we performed a concurrent analysis of the transcriptome and proteome and identified consistent disruption of the protein degradation machinery and spliceosome in samples with USP7 silencing. To further illustrate the utility of the R Shiny framework, we developed DRPPM-EASY-CCLE, a Shiny extension preloaded with the Cancer Cell Line Encyclopedia (CCLE) data. The DRPPM-EASY-CCLE app facilitates the sample querying and phenotype assignment by incorporating meta information, such as genetic mutation, metastasis status, sex, and collection site. As proof of concept, we verified the expression of TP53 associated DNA damage signature in TP53 mutated ovary cancer cells. Altogether, our open-source application provides an easy-to-use framework for omics exploration and discovery.

## 1. Introduction

Multi-omics profiling of cancer patient samples and cell lines is becoming a staple of cancer research [1]. These technologies have a high potential for advancing our understanding of tumor biology and, in turn, reveal novel targets for treatment and diagnosis [2,3]. To date, a brief survey of the existing database reveals more than 500K cancer samples from GEO [4,5] and 90K pre-computed cancer expression data from recount3 [6]. Additionally, there are close to 4K mass spectrometry profiling of cancer patient samples from the Clinical Proteomic Tumor Analysis Consortium (CPTAC) data [7]. Large consortium projects, such as the Cancer Cell Line Encyclopedia (CCLE), have also generated many high-throughput datasets, such as transcript expression, RNA splicing, proteome profiling, drug response, and genetic screening data [8].

With the influx of multi-omics profiling, effective integration of these data remains the bottleneck for omics-driven discovery. The development of a simple user interface that minimizes the need for computational experience is of high interest to the community [9]. Several web-based tools are now available to perform general expression analysis of proteomics (e.g., POMAShiny [10]) and transcriptome data (e.g., TCC-GUI [11], START App [12], and GENAVi [13]). Multi-omics approaches for network analysis (e.g., MiBiOmics [14] and JUMPn [15]) are also available as a Shiny app. Web tools also exist for analyzing large datasets from the Gene Expression Omnibus (GEO) data (e.g., shinyGEO [16], ImaGEO [17]) and the cancer dependency map (e.g., shinyDepMap [18]). However, these applications tend to have limited features for analyzing complex heterogeneous phenotypes in cell lines and patients, such as mutation of genomic drivers, cell line characteristics, sex, or metastasis status. Additionally, none of these tools provides a streamlined pipeline to assess similarities and differences between omics datasets, such as transcriptome and proteome comparisons, or comparisons between mouse and human cancer models.

To address these challenges, we have developed DRPPM-EASY, a Shiny app built with an open-source R programming language that can be run as a local instance or deployed online. Here, our app is divided into two major modules: (1) a one-stop expression analysis for gene expression analysis and (2) an integrative framework for comparing omics data. As a proof of concept, we further implemented an app for querying and automating extraction of sample groupings of CCLE data for downstream analysis. The source code of our application can be downloaded from https://github.com/shawlab-moffitt/DRPPM-EASY-ExprAnalysisShinY (accessed on 1 February 2022).

## 2. Materials and Methods

### 2.1. Module 1. DRPPM-EASY APP Implementation

**Figure 1:**
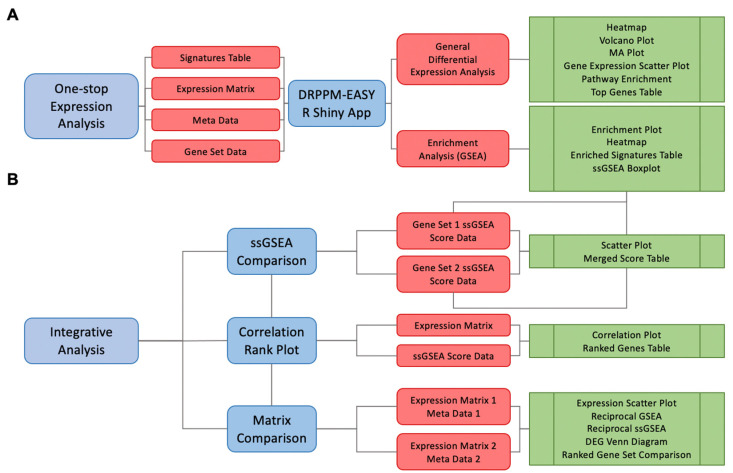
DRPPM-EASY expression analysis pipeline. (**A**) Schematic workflow of DRPPM-EASY. The pipeline takes in input files of an expression matrix, a sample meta-file specifying sample grouping, and a gene set database for GSEA. A GSEA enriched signature table is generated as a preprocessing step, which is used as input to the R Shiny app. The app generates two modes of exploring the data: (1) general differential gene expression analysis and (2) gene set enrichment analysis. The result from the analysis can be downloaded as output tables. (**B**) Schematic of the integrative analysis with three major features for pathway signature comparison. The app has three modes of integrative analysis: (1) scatter plot mode, (2) correlation plot mode, and (3) paired multi-omics analysis.

The DRPPM-EASY app is a Shiny web app built with an open-source R programming language (V.4.1.0). The Shiny framework leverages existing RNA-seq analysis packages to put together a one-stop analysis framework (Figure 1A) for data exploration (Table 1), differential expression analysis (Table 2), and gene set enrichment analysis (Table 3). The data exploration section allows the user to perform unsupervised and supervised hierarchical clustering. Clustering can be further evaluated by different types of distance calculations (i.e., ward, average, complete, centroid) or variable gene ranking strategy (mean absolute deviation or variance). The relative gene expression can be examined across sample groups by a boxplot or scatter plot to examine the gene expression of the positive control associated with the experimental design. Differential gene expression is performed by LIMMA [19] and can be visualized as a volcano plot and MA-plot. The list of differentially expressed genes can be further examined by pathway enrichment analysis (Figure 1A). Finally, the user can perform gene set enrichment analysis (GSEA), which ranks the genes based on signal-to-noise between the user-selected phenotype to examine enriched genes associated with a gene set signature (Figure 1A). A complementary strategy to estimate enrichment scores for individual samples can be performed by single-sample GSEA (ssGSEA) implemented in the GSVA library [20]. Finally, these single-sample enrichment scores can be downloaded as a tab-delimited table or visualized as a boxplot.

### 2.2. Module 2. The DRPPM-EASY-Integration App Implementation

The DRPPM-EASY-Integration provides an explorer for the user to upload normalized RNA expression, proteomic quantification, or ssGSEA scores to evaluate the potential relationship between these features (Figure 1B). These can be evaluated by either a 1:1 scatter plot or 1:n rank of Spearman correlation rho values (Table 4). The integrative app also allows the user to perform concurrent differential expression analysis and integration of two expression matrices, for example, to compare RNA and protein expression matrices. The fold change can be compared between the two datasets (Table 4), and differentially expressed genes can be compared by reciprocal GSEA or ssGSEA. Direct overlap between the differentially expressed genes is shown as a Venn diagram and further compared to existing gene set databases by Fisher’s exact test, Cohen’s kappa score, and the Jaccard index.

### 2.3. Installation and User Guide

The source code and user guide are available for download on the project’s GitHub page. The GitHub page includes the list of individual R packages and their version along with an installation script for all package dependencies.

### 2.4. RNA Sequencing Analysis

USP7 samples were prepared as described in Shaw et al. [21]. Briefly, human T-ALL cell lines Jurkat (ATCC) cells were transduced with USP7 shRNA lentivirus and sorted for GFP positive cells or selected by puromycin. RNA samples were isolated using RNeasy Mini Kit (QIAGEN) and subjected to paired-end 2 × 151 base-pair RNA-seq sequencing (Illumina), 10 Jurkat samples—of which 6 were treated with shRNA and 4 were treated with a scramble RNA—were profiled by RNA-seq. RNA-seq data were processed by a custom pipeline (WRAP, https://github.com/gatechatl/DRPPM_Example_Input_Output/tree/master/WRAP:Wrapper-for-my-RNAseq-Analysis-Pipeline (accessed on 1 August 2021. RNA-seq reads were aligned using the STAR 2.7.1a aligner [22] in the two-pass mode to the human hg38 genome build using gene annotations provided by the Gencode v31 gene models. Read count for each gene was obtained with HT-seq [23]. Reads were normalized to fragments per kilobase million (FPKM) for each gene.

### 2.5. Whole Proteomics Mass Spectrometry and Data Analysis

The 10-plex TMT labeled mass spectrometry experiment was performed with a previously published protocol with slight modification [24,25] (See Supplementary Method, Supplementary Figure S3 for the experimental design). Protein for each sample was digested by trypsin (Promega). The TMT labeled samples were mixed equally, desalted, and fractionated on an offline HPLC (Agilent 1220) using basic pH reverse-phase liquid chromatography (pH 8.0, XBridge C18 column, 4.6 mm × 25 cm, 3.5 μm particle size, Waters). In total, 20 fractions were derived, and the eluted peptides were ionized by electrospray ionization and detected by an inline Orbitrap Fusion mass spectrometer (Thermo Scientific. Waltham, MA, USA). The MS/MS raw files were processed by a tag-based hybrid search engine JUMP [26]. The data were searched against the UniProt human concatenated with a reversed decoy database for evaluating false discovery rate. Searches were performed using a 25 ppm mass tolerance for precursor ions and 25 ppm mass tolerance for fragment ions, fully tryptic restriction with two maximal missed cleavages, three maximal modification sites, and the assignment of *a*, *b*, and *y* ions. TMT tags on lysine residues and N-termini (+229.162932 Da) were used for static modifications, and Met oxidation (+15.99492 Da) was considered as a dynamic modification. MS/MS spectra were filtered by mass accuracy and matching scores to reduce the protein false discovery rate to approximately 1%. Proteins were quantified by summing up reporter ion counts across all matched PSMs using the JUMP software suite [25,26].

### 2.6. Pre-Processing of the GSEA Analysis

To optimize the user experience, we provided a script to pre-generate a GSEA result table (Supplementary Figure S1). The GitHub page contains “Getting Started Scripts”, which allows the user to pre-process GSEA results for downstream table visualization. Enriched signature tables can take a long time to process depending on the number of samples or the size of the GMT file provided by the user. At the top of the script, there are key input parameters, such as file path and name to the expression matrix, metadata, and gene set file, as well as the preferred output file path of the output table(s). Additionally, the getting started scripts include a script to generate an R Data list of the ssGSEA analysis. Large gene sets may require several minutes, so pre-computing can facilitate a better user experience.

## 3. Results

### 3.1. DRPPM-EASY Analysis of RNA-seq and Proteomics Data Use Case 1

**Figure 2:**
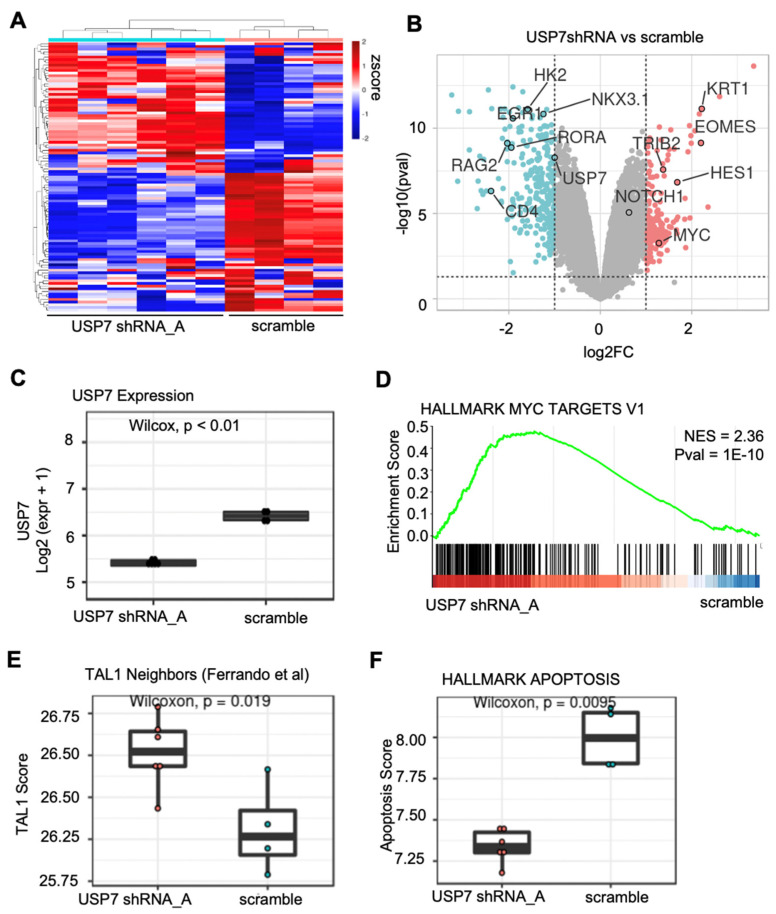
Expression analysis example of RNA-seq data USP7 silenced Jurkat cells. (**A**) Unsupervised clustering of the RNA sequencing data using the top 100 genes ranked based on mean absolute deviation (MAD). (**B**) Differential gene expression analysis comparing USP7 knockdown and scramble. Genes upregulated after USP7 knockdown are shown in red and genes downregulated after USP7 knockdown are shown in blue (USP7-associated targets). (**C**) Boxplot showing the USP7 expression in log2 FPKM. (**D**) Gene set enrichment analysis of MYC targets. (**E**) Boxplot showing the single sample GSVA analysis of the TAL1 gene set. (**F**) Boxplot showing the single sample GSVA analysis of the Hallmark Apoptosis gene set.

We previously identified that USP7 knockdown in T-ALL reduces the activity of E-proteins in a TAL1 dependent manner [21]. To highlight the functions of the DRPPM-EASY application, we re-examined the RNA sequencing profiling data of Jurkat cells after USP7 shRNA silencing. RNA-seq sample grouping was assessed by unsupervised hierarchical clustering (Figure 2A). Notably, altering the clustering methods and the number of (selected) top variables did not change the clustering result, suggesting robust grouping of our data (Supplementary Figure S2). Differential gene expression was then performed by LIMMA and visualized as a Volcano and MA plot. As expected, differential gene expression analysis found downregulated USP7 expression after silencing (Figure 2B,C). Notably, MYC, NOTCH1, TRIB2, and EOMES were upregulated after USP7 knockdown (Figure 2B). In the pathway analysis view, enriched pathways can be examined with preloaded gene sets from MsigDB, cell marker, and L1000 drug response. By GSEA and single-sample GSEA, we found USP7 knockdown upregulated with MYC and TAL1 associated targets (Figure 2D,E) and found downregulated apoptotic gene signature from the Hallmark database (Figure 2F). Overall, the RNA-seq analysis supports our previous finding that USP7 is implicated in the negative regulation of TAL1-dependent leukemia growth [21].

**Figure 3:**
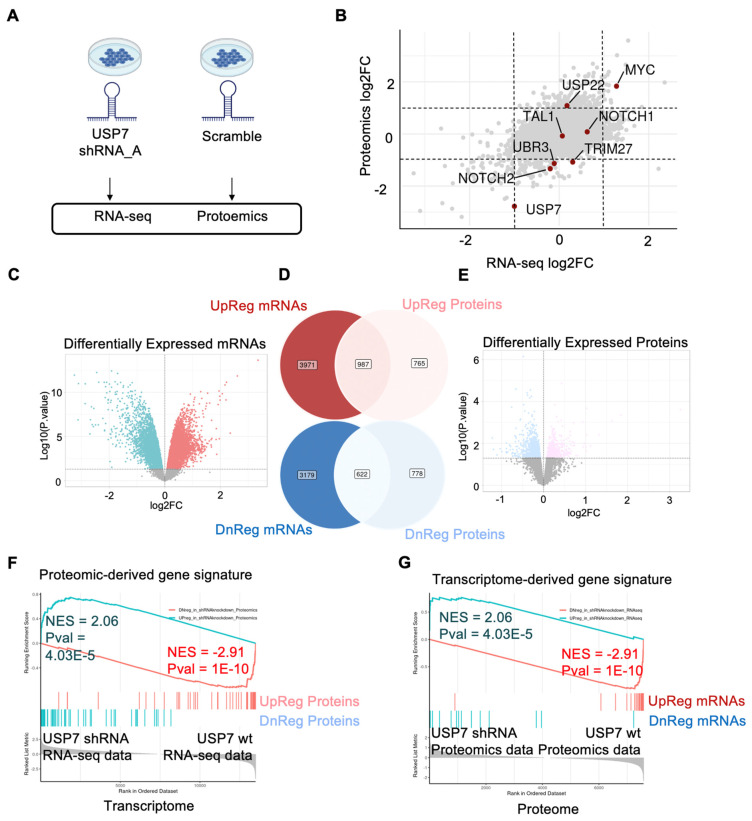
Integrated analysis example of proteomics and transcriptomics USP7 silenced Jurkat cells. (**A**) Jurkat samples treated with USP7 shRNA and scramble were profiled by RNA sequencing and TMT mass spectrometry. (**B**) The log2 fold change from the differential expression analyses is plotted. Positive log2FC indicates upregulated expression after USP7 silencing. Negative log2FC indicates downregulated expression after USP7 knockdown. Dotted line indicates the −1 and 1 log2FC cutoff. (**C**) Upregulated and downregulated gene signatures derived from differentially expressed mRNAs. (**D**) Venn diagram of genes differentially upregulated (top panel) and downregulated (bottom panel) in the transcriptome (left) and proteome (right). (**E**) Up-regulated and downregulated gene signatures derived from differentially expressed proteins. (**F**,**G**) Reciprocal GSEA of differentially expressed genes derived from the transcriptome and examined in the proteomics data (**F**)**.** Similarly, differentially expressed proteins were first derived then examined in the transcriptome data by GSEA (**G**).

Next, tandem-mass-tagged proteomics profiling was performed on the same set of samples with RNA-seq profiling (Figure 3A; Supplementary Figure S3). A joint analysis of the transcriptome and proteome data was carried out by the DRPPM-EASY-Integration pipeline, identifying genes with altered protein abundance and unaltered mRNA levels, such as TRIM27, NOTCH2, UBR3, and USP22 (Figure 3B). Consistent with our previous observation, TRIM27, a known target of USP7 [27], observed decreased protein abundance in T-ALL cell lines with a haploinsufficient *USP7* [21]. The altered abundance of UBR3 and USP22 suggests an altered ubiquitin ligase network. Furthermore, our result suggests that USP7 loss-of-function alters NOTCH2 protein abundance. Of note, NOTCH1 [28] protein abundance was unaltered after USP7 knockdown (Figure 3B). Thus, the precise mechanism of USP7 to drive the NOTCH association leukemia signature will need to be carefully examined in future studies.

The DRPPM-EASY-Integration includes features assessing the consistency between two datasets. Using the RNA-seq and proteomic data as proof of concept, DRPPM-EASY-Integration found 987 genes consistently upregulated, and 622 genes consistently downregulated in both datasets (Figure 3C–E). A connectivity map-inspired strategy [29,30] was applied to compare the consistency between the two datasets using reciprocal enrichment. Specifically, differential expressed genes in one dataset was used to derive a gene signature for GSEA to test in the other dataset. For example, differentially expressed proteins (Figure 3F) were applied as a GSEA gene set and tested for enrichment in the transcriptome data (Figure 3G). Similarly, gene sets derived from differentially expressed transcripts (Figure 3C) were tested for enrichment in the proteome data (Figure 3H). We then compared the significance of the overlapping differentially expressed genes against other pathway databases, such as Hallmark and KEGG. The overlap was evaluated by Fisher’s exact test, Cohen’s kappa, and Jaccard index. Consistently, the RNA and protein were most significantly overlapped compared to other gene sets. Moreover, the spliceosome and ubiquitin-mediated proteolysis pathways from KEGG and the unfolded protein response and MYC pathway from Hallmark were consistently enriched in both datasets (Supplementary Figure S3B,C; Supplementary Tables S1 and S2).

### 3.2. DRPPM-EASY-CCLE Use Case 2

**Figure 4:**
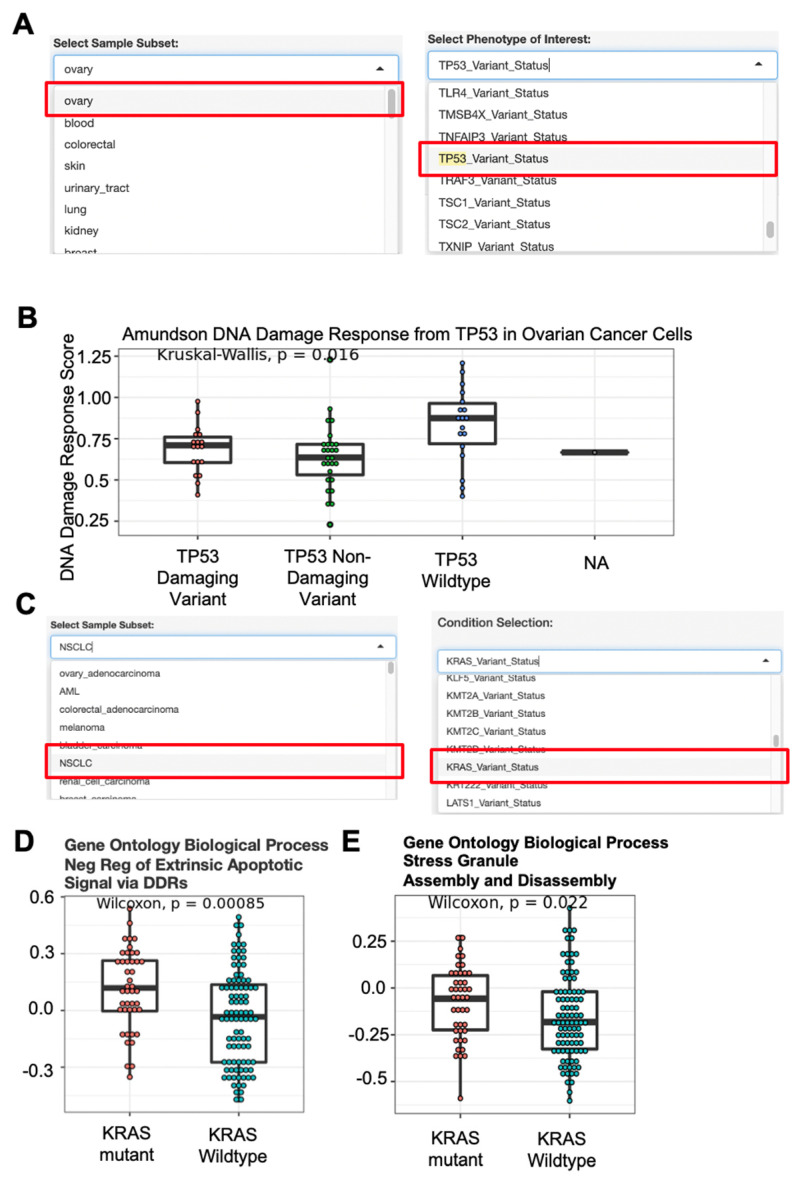
Use case analysis example of CCLE Expression data. (**A**) Drop-down menu selection of sample cohort and sample phenotype characteristic. CCLE ovary samples and TP53 mutation status were selected from the drop-down menu option. (**B**) Single-sample GSEA analysis of genes defining the DNA damage response by Amundson et al. Analyzed samples were selected from the drop-down menu from (**A**). (**C**) Drop-down menu selection of sample cohort and sample phenotype characteristic. CCLE non-small cell lung cancer samples and phenotype associated with the KRAS mutation status were selected from the drop-down menu option. (**D**) Single sample GSEA analysis of genes negatively regulating the DNA damage response. (**E**) Single sample GSEA of genes defining the stress granule assembly and disassembly. Gene sets were compiled from Biological Pathways from the Gene Ontology database (GOBP). Analyzed samples were selected from the drop-down menu from (**C**).

To further illustrate the DRPPM-EASY functionality, we developed DRRPM-EASY-CCLE, an extended app with features to select samples from the Cancer Cell Line Encyclopedia (CCLE) data. The app is preloaded with 1379 CCLE samples spanning 37 lineages, 96 lineage sub-types, and 33 diseases. For the genetic characterization, 299 cancer drivers [31] were selected and further divided based on the damaging and non-damaging variant status from DepMap [32] (see Supplementary Table S3 for the complete phenotype categories). As an example, we extracted ovary cancer cell lines and performed expression analysis comparing *TP53* mutation status to its wild-type counterpart (Figure 4A). In *TP53* mutated ovary cancer cells, we found a decreased DNA damage response gene signature (Figure 4B), thereby solidifying the role of *TP53* loss-of-function for regulating DNA damage in these ovarian cancer cells.

Previously, KRAS was found to be frequently mutated in non-small cell lung cancer (NSCLC) and is associated with drug resistance [33]. Thus, we analyzed NSCLC cell lines and compared KRAS mutation status to its wild-type counterpart (Figure 4C). By pathway analysis, the MsigDB defined KRAS signature was consistently upregulated in our KRAS mutated samples (Supplementary Figure S4A). Interestingly, top pathways enriched in the KRAS mutated samples are associated with an anti-apoptosis signature (Supplementary Figure S4B). By ssGSEA, amplified expression in KRAS mutated NSCLC cells were enriched with genes that negatively regulate apoptosis (Figure 4D) and upregulating genes that associated with stress granule assembly and disassembly (Figure 4E), which is a dynamic process fundamental to surviving under stress [34]. Interestingly, oncogenic KRAS-driven stress granules were previously identified in pancreatic and colorectal adenocarcinoma [35]; thus, our result suggests a similar stress response in NSCLC cells.

To further expand our functionality for exploring these large project data, we have also implemented features that enable users to upload their own expression matrix to perform an integrative analysis in CCLE and lung squamous cell carcinoma CPTAC datasets https://github.com/shawlab-moffitt/DRPPM-EASY-LargeProject-Integration (accessed on 1 February 2022) (Supplementary Figures S5A–C). Altogether, our framework provides a user-friendly environment to categorize the samples for downstream analysis with a high potential for novel discovery.

## 4. Discussion

An effective method for visualization and data analysis is key to the analysis of multi-omics data that captures the molecular processes of cancer initiation and progression. Several Shiny apps have been published to date and can be categorized into the following three categories: (1) tools that focus on pairwise differential expression and biomarker discovery (e.g., POMAShiny 10], TCC-GUI [11], and START App [12]), (2) tools that perform pathway and network analysis (e.g., iOmics [14] and JUMPn [15]), and (3) tools that facilitate the query of large datasets, such as from public repositories or consortium deposited datasets and deposited expression data (e.g., shinyGEO [16], ImaGEO [17], and GENAVi [13]). While numerous web tools have been developed thus far, there is a lack of tools that directly address challenges associated with multi-data integration, such as evaluating the consistency between omics datasets.

Here, we developed an interactive software tool, DRPPM-EASY, that allows users to perform complex omics data integration in both small (pairwise comparison) and large (consortium) projects. DRPPM-EASY puts together an interactive flexible interface that enables the exploration of biomarkers and enriched pathways across multiple datasets. DRPPM-EASY can perform routine gene analysis, such as hierarchical clustering, differential gene expression, pathway analysis, GSEA, and ssGSEA. Additionally, DRPPM-EASY can perform a joint analysis of two expression datasets. As an example, we have highlighted the application’s ability to evaluate the consistency between transcriptome and protein datasets. This is made possible by deriving a gene set feature in one dataset (i.e., transcriptomics), which is applied in the GSEA analysis of the other dataset (i.e., proteomics). DRPPM-EASY can be easily adapted for large consortium data, which we highlight as an example in CCLE cancer cell lines and lung squamous cell carcinoma CPTAC proteome data. Finally, to further expand the utility of our tool, the user can upload their own expression data and use it to compare against CCLE cell lines and lung squamous cell carcinoma proteome data. One major limitation of our application requires the user to normalize their gene expression matrix prior to using our application. Existing pipelines are available to streamline the normalization procedure, such as Shiny-Seq [36]. A normalization procedure will be included in future updates of our application.

Finally, the ability to run the application with a user interface on a local desktop reduces the need for computational domain knowledge of expression analysis. The DRPPM-EASY application can be set up on the server in real-time, enabling collaborative discussion on potential hypotheses derived from the high-throughput data. Our tool also ensures reproducibility of the data analysis, which is one of the most significant issues in omics research [37]. While the current application is highlighted to work in RNA-seq and proteomics data, our framework could easily be adapted to incorporate drug response, genetic screening, or splicing associated features in future versions of our application. Thus, we believe DRPPM-EASY will be a useful and valuable tool for the biomedical research community.

## Figures and Tables

**Table 1:** Data Exploration Module.

	App Function	Description
E1	Unsupervised Heatmap	Top variable gene selection Expression data is log2 transformed then z-normalized User-specified clustering method
E2	Scatter Plot	User selects two genes of interest Expression values compared via interactive scatter plot (log2 transformation is optional)
E3	Custom Heatmap	Visualize user-selected genes and samples Expression data is log2 transformed and z-normalized User-specified clustering method
E4	Box Plot	Gene expression in each group are shown Expression values are log2 transformed Comparing groups for statistical differences

**Table 2:** Differential Expression Analysis Module.

	App Function	Description
DEA1	Volcano Plot	User selects comparison groups Differential gene expression analysis with LIMMA Up- and downregulated differentially expressed genes determined with user input
DEA2	MA Plot	User selects comparison groups Differential gene expression analysis with LIMMA Up- and downregulated differentially expressed genes determined with user input
DEA4	Pathway Enrichment Analysis	User selects comparison groups and gene set/pathway Differential gene expression analysis with LIMMA Pathway enrichment analysis using enrichR

**Table 3:** Gene Set Enrichment Analysis Module.

	App Function	Description
GA1	Enrichment Plot	User selects comparison groups Signal-to-noise ranking performed on expression data GSEA function performed with chosen gene set
GA2	Gene Expression Heatmap	User selects comparison groups Signal-to-noise ranking performed on expression data GSEA function performed with chosen gene set Expression data log2 transformed and scaled Genes from chosen gene set displayed in the heatmap
GA3	GSEA Summary Table	Displays user pre-generated enriched signatures table
GA4	Generate Summary Table	GSEA function performed on expression data with user input GMT file Enriched signatures table produced is displayed
GA5	ssGSEA Boxplots	User-selects gene set and single-sample GSEA method Comparing groups for statistical differences

**Table 4:** Integrative Analysis.

	App Function	Description
IA1	Scatter Plot Comparison	User input features are merged and plotted Samples are colored based on metadata type
IA2	Correlation Rank Plot	Assessing the relationship between ssGSEA score and gene expression performed Correlation can be performed as Spearman, Pearson, or Kendall Correlation values plotted by rank from lowest to highest
IA3	Matrix Comparison File Upload	Upload two expression matrices and two metadata files
IA4	Log2FC Comparison Scatter Plot	Differential gene expression analysis with LIMMA performed on both matrices Log2 fold change values subset and difference between matrices calculated Expression data displayed as scatter plot
IA5	Reciprocal GSEA	Differential gene expression analysis with LIMMA Four gene sets derived differentially expressed genes (two upregulated, and two downregulated gene set) GSEA performed on the reciprocal data
IA6	Reciprocal ssGSEA	Differential gene expression analysis with LIMMA Four gene sets derived differentially expressed genes (two upregulated, and two downregulated gene set) ssGSEA performed on the reciprocal data
IA7	Venn Diagram	Differential gene expression analysis with LIMMA Overlapping differentially expressed genes Perform Fisher’s exact test. Calculate Cohen’s kappa, and Jaccard index to compare between the two matrix and across user selected pathways.

## Data Availability

The developed software and processed data can be downloaded from the following GitHub page https://github.com/shawlab-moffitt/DRPPM-EASY-ExprAnalysisShinY (accessed on 1 February 2022).

## Supplementary Materials

The following supporting information can be downloaded at: https://www.mdpi.com/article/10.3390/biology11020260/s1, Supplementary Method. Supplementary Table S1. KEGG Pathways Jointly Enriched in the Transcriptome and Proteome. Supplementary Table S2. Hallmark Pathways Jointly Enriched in the Transcriptome and Proteome. Supplementary Table S3. CCLE Sample Meta-Information. Supplementary Figure S1. Schematic of the GSEA pre-processing. Supplementary Figure S2. Unsupervised hierarchical clustering of Jurkat samples after USP7 knockdown. Supplementary Figure S3. Experimental design of the total proteome profiling of the USP7 knockdown experiment. Supplementary Figure S4. Pathway enrichment analysis of genes differentially upregulated in KRAS mutated samples in NSCLC. Supplementary Figure S5. Screen shot showing the user option to upload user data in the DRPPM-Large Project Integration.
