# Supplementary material for: DRPPM-EASY: A Web-Based Framework for Integrative Analysis of Multi-Omics Cancer Datasets
Source: Biology (Basel). 2022 Feb 8;11(2):260. doi: 10.3390/biology11020260 (PMC8869715; doi:10.3390/biology11020260)
Supplement: Supplementary file 1 [file biology-11-00260-s001.zip › Supplementary_Figures.pdf]

Supplementary Figure S1.

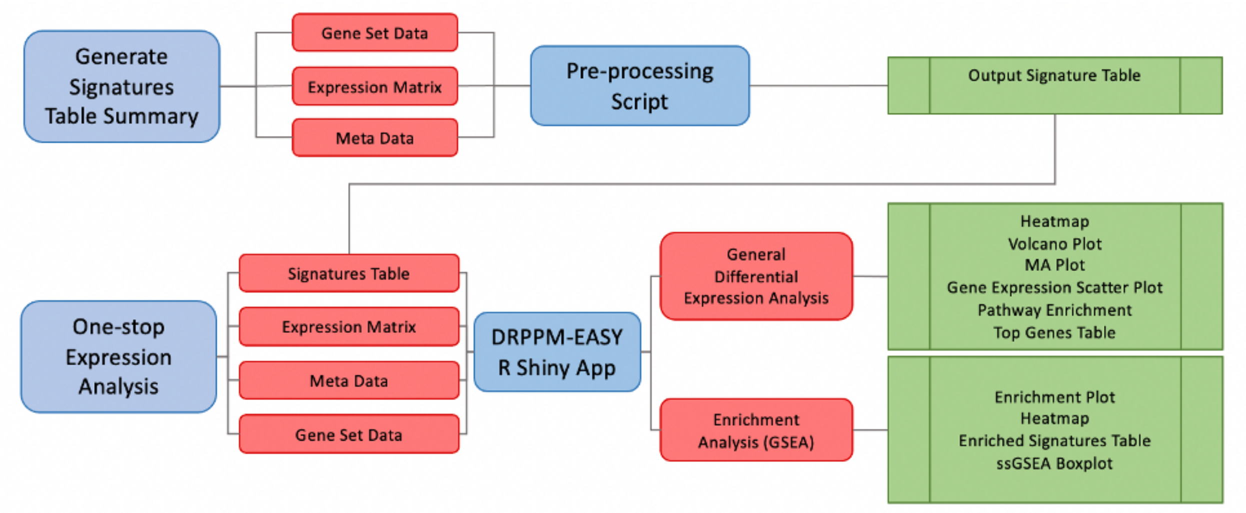

Supplementary Figure S1. Schematic of the GSEA pre-processing.

Supplementary Figure S2.

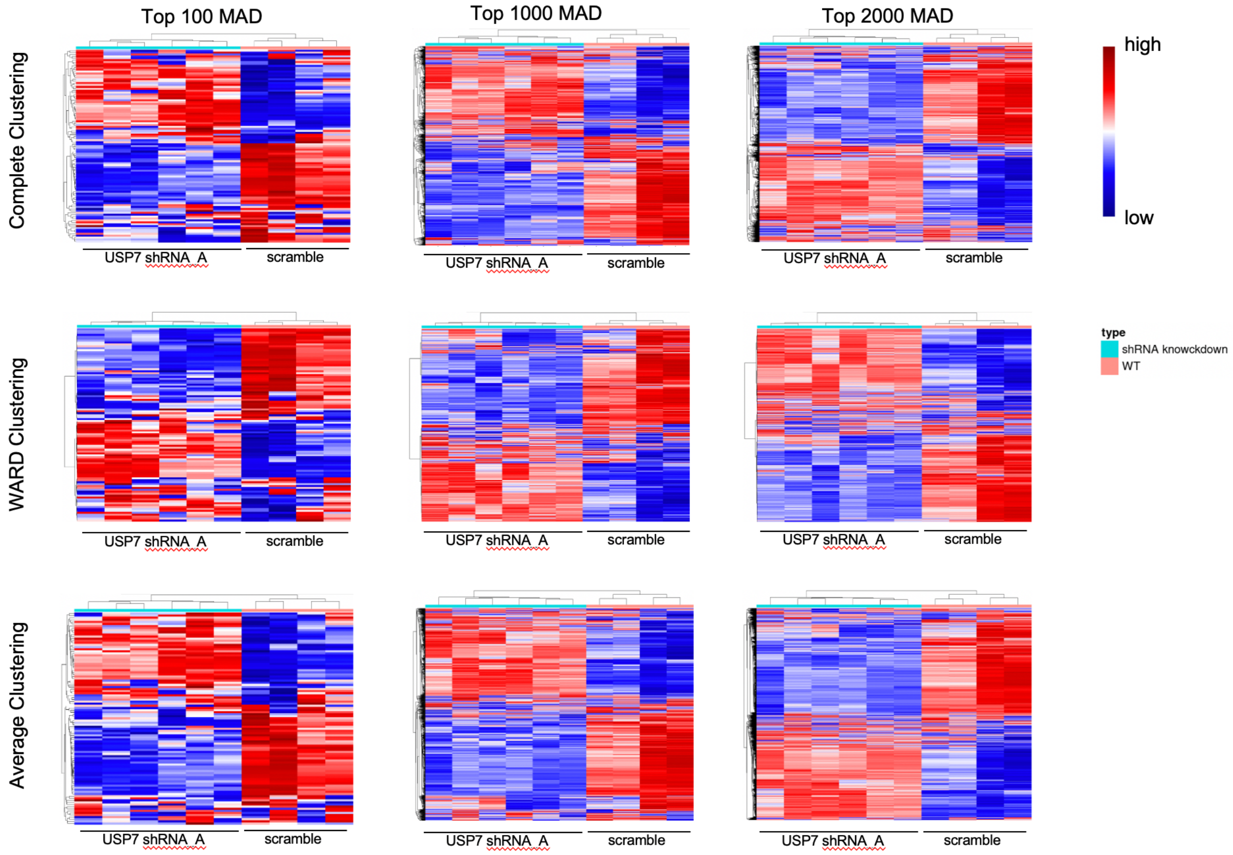

Supplementary Figure S2. Unsupervised hierarchical clustering of Jurkat samples after USP7 KD. Each row represents distinct clustering algorithms: Complete, WARD, and Average. Each column represents different number of variable genes selected by Mean Absolute Deviation (MAD).

Supplementary Figure S3.

A

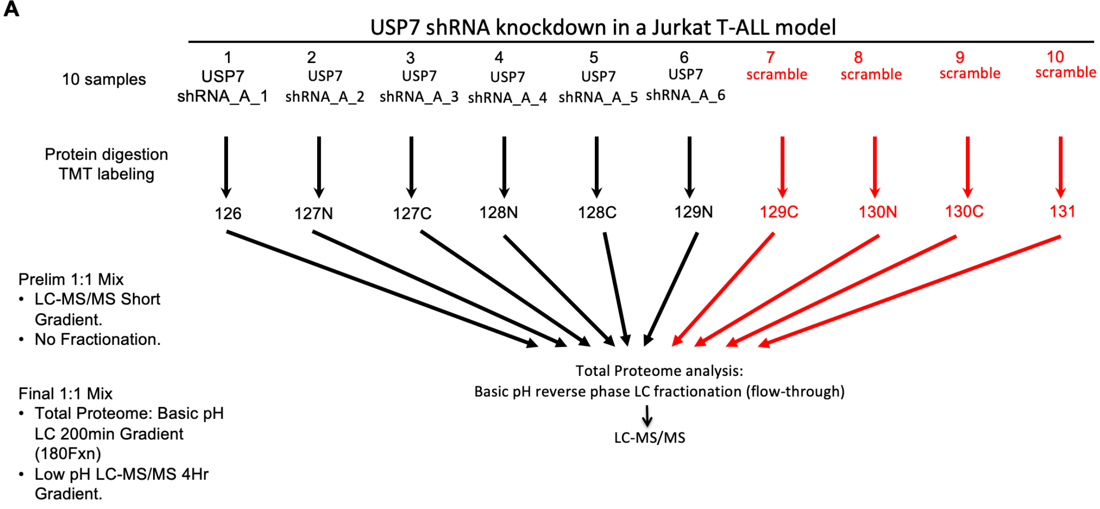

B

GeneSets	Top Enriched Pathways
KEGG_SPLICEOSOME	1
KEGG_RNA_POLYMERASE	2
KEGG_PYRIMIDINE_METABOLISM	3
KEGG_LYSOSOME	4
KEGG_HUNTINGTONS_DISEASE	5
KEGG_TGF_BETA_SIGNALING_PATHWAY	6
KEGG_PURINE_METABOLISM	7
KEGG_CELL_CYCLE	8
KEGG_UBIQUITIN_MEDIATED_PROTEOLYSIS	9
KEGG_P53_SIGNALING_PATHWAY	10

C

GeneSets	Top Enriched Pathways
HALLMARK_UV_RESPONSE_UP	1
HALLMARK_INTERFERON_GAMMA_RESPONSE	2
HALLMARK_G2M_CHECKPOINT	2
HALLMARK_UNFOLDED_PROTEIN_RESPONSE	4
HALLMARK_MYC_TARGETS_V2	5
HALLMARK_ALLOGRAFT_REJECTION	6
HALLMARK_E2F_TARGETS	7
HALLMARK_MYC_TARGETS_V1	8
HALLMARK_ESTROGEN_RESPONSE_EARLY	9
HALLMARK_INTERFERON_ALPHA_RESPONSE	10

Supplementary Figure S3. A) Experimental design of the total proteome profiling of the USP7 knockdown experiment. B, C) Upregulated pathway ranked based on Jaccard index, Odds Ratio, and Cohen's Kappa in the KEGG database (B) and the HALLMARK database (C).

Supplementary Figure S4.

A

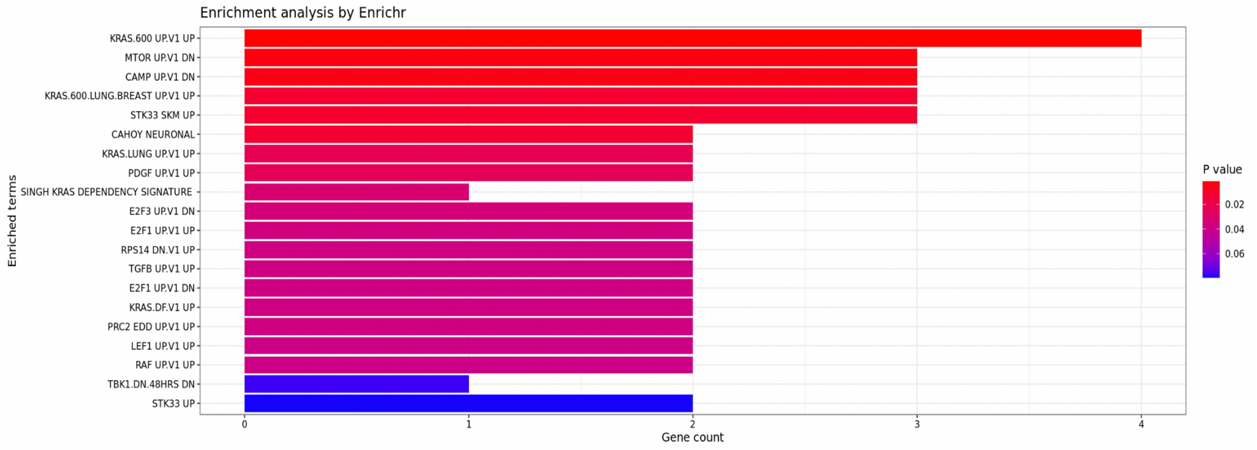

B

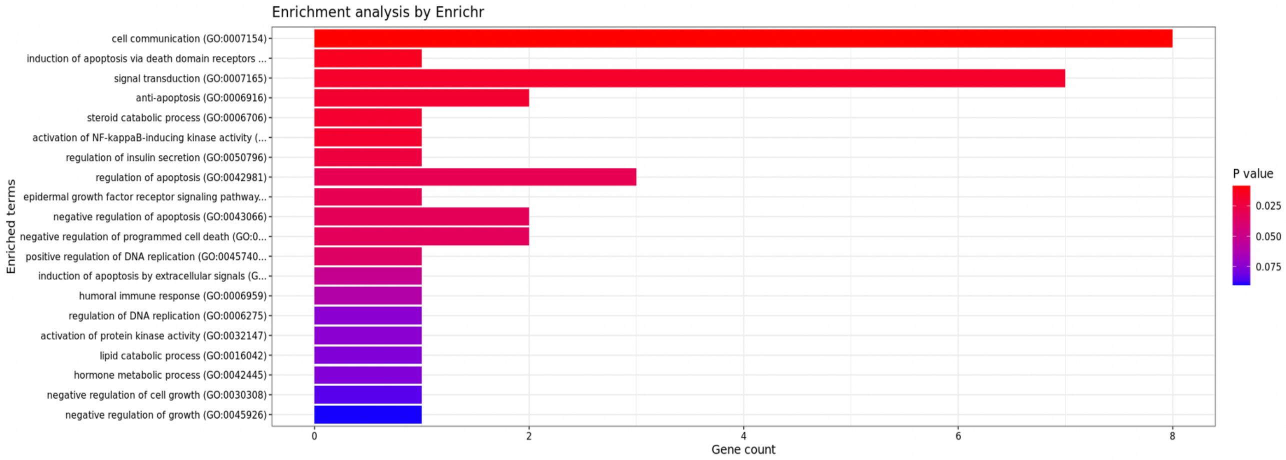

Supplementary Figure S4. Pathway enrichment analysis of genes differentially upregulated in KRAS mutated samples in NSCLC. A) Pathway analysis using the MsigDB Oncogenic Signature database. B) Pathway analysis using the GO Biological Process Gene Signatures.

Supplementary Figure S5.

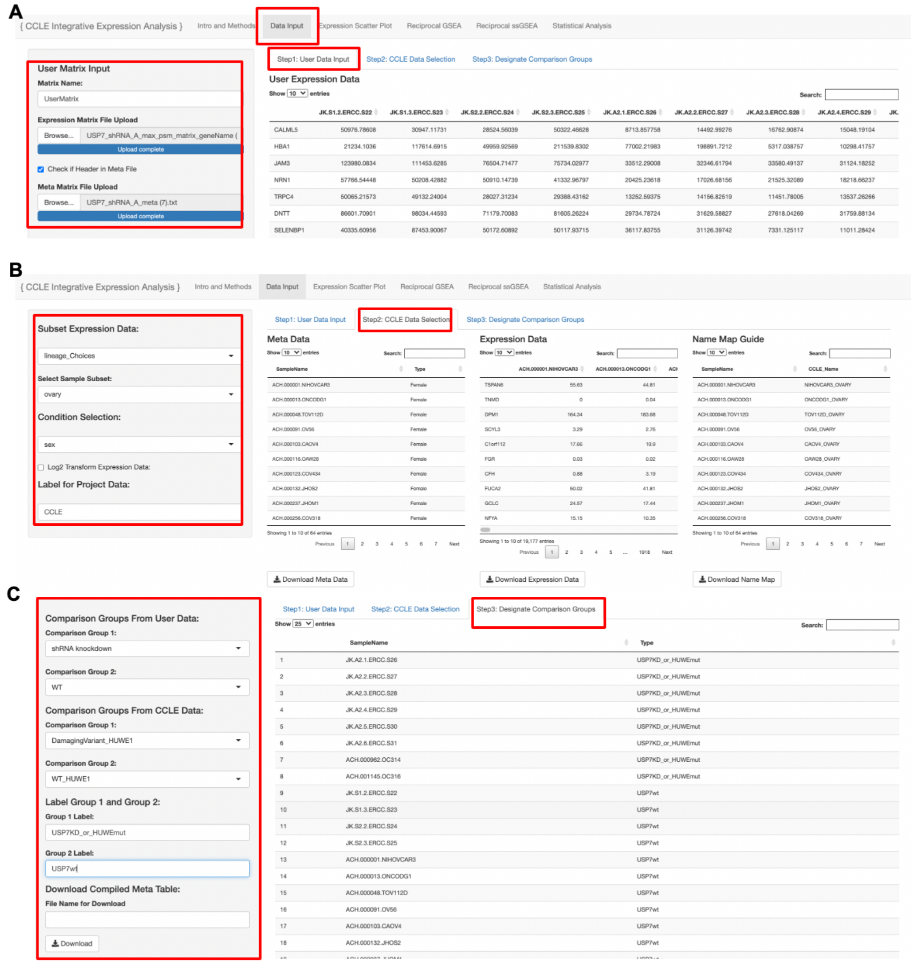

Supplementary Figure S5. Screen shot showing the user option to upload user data in the DRPPM-Large Project Integration function. (CCLE example). **A)** Step 1. The user can upload a matrix and its associated sample meta-information file. **B)** Step 2. The user will select the CCLE sample cohort to analyze and select the phenotype for the group comparison. **C)** Step3. Harmonizes the sample grouping between the two datasets.
