# Supplementary material for: DRPPM-EASY: A Web-Based Framework for Integrative Analysis of Multi-Omics Cancer Datasets
Source: Biology (Basel). 2022 Feb 8;11(2):260. doi: 10.3390/biology11020260 (PMC8869715; doi:10.3390/biology11020260)
Supplement: Supplementary file 1 [file biology-11-00260-s001.zip › Supplementary_Methods.pdf]

Supplementary Methods

Whole proteomics Protein Digestion and Peptide Isobaric Labeling by Tandem Mass Tags (TMT)

Cell pellets were extracted in lysis buffer (50 mM HEPES, pH 8.5, 8 M urea, and 0.5% sodium deoxycholate). The protein concentration of the lysates was determined by a Coomassie-stained short gel with bovine serum albumin (BSA) as a standard (1). 200μg of protein for each sample was digested with LysC (Wako) at an enzyme-to-substrate ratio of 1:100 (w/w) for 2h in the presence of 1mM DTT. Next, the samples were diluted to a final 2M Urea concentration with 50mM HEPES (pH 8.5), and further digested with Trypsin (Promega) at an enzyme-to-substrate ratio of 1:50 (w/w) for at least 3h. The peptides were reduced by adding 1mM DTT for 30 min at room temperature (RT) followed by alkylation with 10mM iodoacetamide (IAA) for 30 min in the dark at RT. The unreacted IAA was quenched with 30mM DTT for 30 min. Finally, the digestion was terminated and acidified by adding trifluoroacetic acid (TFA) to 1%, desalted using C18 cartridges (Harvard Apparatus) and dried by speed vac. The purified peptides were re-suspended in 50mM HEPES (pH 8.5), labeled with 10-plex Tandem Mass Tag (TMT) reagents (Thermo Scientific) following the manufacturer's recommendation.

Whole proteomics two dimensional HPLC and Mass Spectrometry

The TMT-10plex labeled samples were mixed equally, desalted, and fractionated on an offline HPLC (Agilent 1220) using basic pH reverse-phase liquid chromatography (pH 8.0, XBridge C18 column, 4.6 mm x 25 cm, 3.5 μm particle size, Waters). The fractions were dried and resuspended in 5% formic acid and analyzed by acidic pH reverse phase LC-MS/MS analysis. The peptide samples were loaded on a nanoscale capillary reverse phase C18 column (New objective, 75 μm ID x ~15 cm, 1.9 μm C18 resin from Dr. Maisch GmbH) by an HPLC system (Thermo Ultimate 3000) and eluted by a 60 min gradient. The eluted peptides were ionized by electrospray ionization and detected by an inline Orbitrap Fusion mass spectrometer (Thermo Scientific). The mass spectrometer is operated in data-dependent mode with a survey scan in Orbitrap (60,000 resolution, 2 X 10^5 AGC target, and 50 ms maximal ion time) and MS/MS high-resolution scans (60,000 resolution, 1 X 10^5 AGC target, 150 ms maximal ion time, 38 HCD normalized collision energy, 1 *m/z* isolation window, and 15 s dynamic exclusion).
